# Biocatalysis of Fucodian in *Undaria pinnatifida* Sporophyll Using *Bifidobacterium longum* RD47 for Production of Prebiotic Fucosylated Oligosaccharide

**DOI:** 10.3390/md17020117

**Published:** 2019-02-14

**Authors:** Jeong Min Lee, So Young Oh, Tony V. Johnston, Seockmo Ku, Geun Eog Ji

**Affiliations:** 1 Department of Food and Nutrition, Research Institute of Human Ecology, Seoul National University, Seoul 08826, Korea; helloseas@sk.com (J.M.L.); soandho@snu.ac.kr (S.Y.O.); 2 Fermentation Science Program, School of Agriculture, College of Basic and Applied Sciences, Middle Tennessee State University, Murfreesboro, TN 37132, USA; tony.johnston@mtsu.edu; 3 Research Center, BIFIDO Co., Ltd., Hongcheon 25117, Korea

**Keywords:** fucosylated oligosaccharide, marine polysaccharide, l-fucose, fucoidan, *Undaria pinnatifida*, prebiotics, *Bifidobacterium*

## Abstract

Fucosylated oligosaccharide (FO) is known to selectively promote the growth of probiotic bacteria and is currently marketed as a functional health food and prebiotic in infant formula. Despite widespread interest in FO among functional food customers, high production costs due to high raw material costs, especially those related to fucose, are a significant production issue. Therefore, several actions are required before efficient large-scale operations can occur, including (i) identification of inexpensive raw materials from which fucosylated oligosaccharides may be produced and (ii) development of production methods to which functional food consumers will not object (e.g., no genetically modified organisms (GMOs)). *Undaria pinnatifida*, commonly called Miyeok in Korea, is a common edible brown seaweed plentiful on the shores of the Korean peninsula. In particular, the sporophyll of *Undaria pinnatifida* contains significant levels of l-fucose in the form of fucoidan (a marine sulfated polysaccharide). If the l-fucose present in *Undaria pinnatifida* sporophyll was capable of being separated and recovered, l-fucose molecules could be covalently joined to other monosaccharides via glycosidic linkages, making this FO manufacturing technology of value in the functional food market. In our previous work, β-galactosidase (EC 3.2.2.23) from *Bifidobacterium longum* RD47 (*B. longum* RD47) was found to have transglycosylation activity and produce FO using purified l-fucose and lactose as substrates. In this research, crude fucodian hydrolysates were separated and recovered from edible seaweed (i.e., *U. pinnatifida* sporophyll). The extracted l-fucose was purified via gel permeation and ion exchange chromatographies and the recovered l-fucose was used to synthesize FO. *B. longum* RD47 successfully transglycosilated and produced FO using l-fucose derived from *Undaria pinnatifida* and lactose as substrates. To the best of our knowledge, this is the first report of synthesized FO using *Bifidobacterium* spp.

## 1. Introduction

l-Fucose (CAS#=2438-80-4; C_6_H_12_O_5_) is classified as hexose deoxy sugar with a hydroxyl group replaced by a hydrogen atom at the 6-position carbon [1,2]. l-Fucose is abundant in brown algae such as *Fucus, Laminaria, Sargassum*, and *Undaria* spp., as a major constituent of fucoidan [2]. Fucoidan is a group of marine sulfated polysaccharides from the cell wall matrix of brown algae containing large proportions of l-fucose, sulfate-fucose, and galactose together with minor sugars [3]. The chemical structures of fucoidans in brown algae vary by species [2]. According to Mak et al. [4], fucoidan contains l-fucose as a major carbohydrate. Recent studies have shown the existence of three types of algal fucoidans: one consisting of (1→3)-α-l-fucose residues [5,6], a second containing alternating (1→3)- and (1→4)-α-l-fucose residues [7,8], and a third built out of (1→3)- or (1→3, 1→4)-linked α-l-fucose and β-d-galactose residues [9]. Among the various brown algae, *Undaria pinnatifida*, commonly called Miyeok in Korea, is a common edible brown seaweed plentiful on the shores of the Korean peninsula. In particular, the sporophyll of *Undaria pinnatifida* contains relatively higher amounts of fucoidan relative to other parts of the algae [9,10].

Fucose is also found in human milk. Human milk oligosaccharides are known to promote growth and provide protection against pathogens in the intestinal tracts of infants [11]. Approximately 200 neutral and acidic oligosaccharide species with a high degree of fucosylation have been identified in pooled human milk samples. These oligosaccharides are terminated by fucose and exhibit fucosylation rates between 50% and 70% [12,13]. Among the various probiotic cell strains, fucosylated oligosaccharide (FO) can promote the selective growth of *Bifidobacterium* spp., a well-known beneficial bacterium in the large intestine [14]. Also, FO can inhibit the adhesion of pathogens on epithelial cells [15].

Multiple scholars have reported that microbial galactosidases reveal transglycosyl activities toward l-fucose [16,17]. In our previous work, crude enzyme extracts from *B. longum* RD47 were found to have transglycosylation activities that produced FO using l-fucose with lactose as a substrate [18]. However, commercially available l-fucose is prohibitively expensive, limiting its use in producing FO in or for the food industry [19]. In this study, we successfully extracted l-fucose from fucoidan of *U. pinnatifida* sporophyll and used this extract as the raw material for synthesizing FO through *B. longum* RD47 biocatalysis.

## 2. Results and Discussion

### 2.1. Extraction and Purification of Monosaccharides from U. pinnatifida Sporophyll

The diverse functional materials extracted from seaweeds have attracted great attention among food companies due to their potential as functional foods. The market for these substances is estimated in the billions of dollars [20]. Among them, fucoidan is one of the heterogeneous sulfated complex sugars commonly existing in brown algae. However, the structural characteristics of fucoidan differ in biological activity, depending on the geographical location of the seaweed in which it is found. This is related to the degree of sulphation and the types of monosaccharides within the fucoidan. To evaluate the potential for utilization of *U. pinnatifida* fucoidan, we characterized it through both quantitative and qualitative analyses.

**Figure 1:**
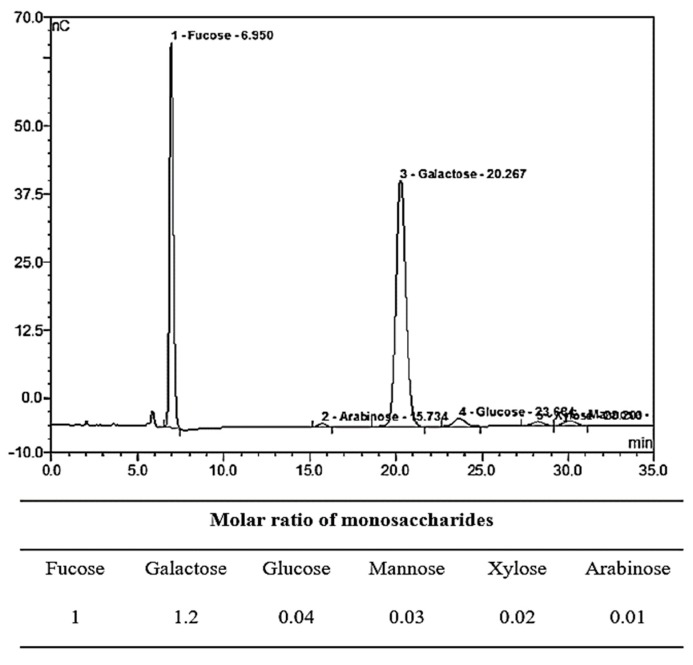
Bio-LC analysis of monosaccharide composition in the *U. pinnatifida* sporophyll hydrolysate, including the following: (1) fucose; (2) arabinose; (3) galactose; (4) glucose; (5) xylose; and (6) mannose.

Crude algal monosaccharides were isolated from *U. pinnatifida* sporophyll via acid-catalyzed hydrolysis, and the recovered hydrolysates were applied to Bio-LC to evaluate monosaccharide compositions. Each monosaccharide was quantified by comparing the peak area of the sample sugar to that of a standard monosaccharide of known concentration (Figure 1). The molar ratio of each monosaccharide from the sample was then determined.

From each 100 g sample of dried *U. pinnatifida* sporophyll, 5.51 ± 1.3 g of monosaccharide was obtained with a dry mass yield of 5.5%. The yield of monosaccharides from *U. pinnatifida* sporophyll was similar to the ≥3.2% yields previously reported [4,21]. The monosaccharide composition of the purified fucoidan was shown to be galactose, fucose, glucose, mannose, xylose, and arabinose. The major compounds were galactose and fucose with a molar ratio of 1.2:1. The molar ratio of galactose and fucose from *U. pinnatifida* sporophyll was consistent with a previously reported 1.1:1 ratio [22]. Because our sample was found to contain a sufficient amount of fucose, we conducted further studies to evaluate whether *U. pinnatifida* hydrolysates could be used for oligosaccharide production via enzymatic glycosylation. *U. pinnatifida* hydrolysates potentially contain impurities (e.g., sulfate, phosphate, ions, and organic acids) in addition to monosaccharides, which act as enzyme inhibitors. Gel permeation chromatography and ion-exchange chromatography have been shown to not alter the structure of saccharides, so these techniques were used to separate the sugar substances from the acid hydrolysates. Those chromatographic fractions containing l-fucose were further applied to generate FO through transglycosylation of *B. longum* RD47 β-galactosidase.

### 2.2. Synthesis of Fucosylated Oligosaccharide Using β-Galactosidase of B. longum RD47

β-Galactosidase is an enzyme commonly used to prevent lactose crystallization in frozen dairy products via lactose hydrolysis into glucose and galactose. In addition to sugar hydrolysis, β-galactosidase also demonstrates transglycosylation properties when interacting with several simple sugars and recently has been used to produce a variety of oligosaccharides in the lab and at the pilot scale level. Efforts have also been made to produce FO using l-fucose and β-galactosidase biocatalysis. However, the majority of researchers did not specify the origin of the raw materials used in l-fucose production. Finally, several groups have used non-GRAS microorganisms (e.g., *E. coli* and *Bacillus* spp.) or genetically modified microorganisms to produce the β-galactosidase used in the production of FO [23,24,25].

Even though numerous institutions and 110 Nobel laureates have confirmed the safety of genetically modified organisms (GMOs), food consumers continue to be suspicious and non-supportive of them [26,27]. Moreover, health and functional food consumers are willing to pay more to purchase non-GMO foods [28,29,30]. As FO will most likely be an ingredient in functional foods, infant formulas, and pre/probiotic products, the use of GMOs or non-GRAS microorganisms in their production will be a challenge for product approval, at best. Using enzymes naturally produced by probiotic cell (e.g., *Bifidobacterium* and *Lactobacillus* spp.) biocatalysis to produce bioactive substances is thus becoming increasingly important in these food industries. In our previous work, the β-galactosidase of *B. longum* RD47, which has demonstrated transglycosylation activity, aided in FO production using l-fucose and lactose. In the process of transglycosylation, l-fucose acts as an acceptor in the formation of FO [18]. Therefore, l-fucose extracted and purified from *U. pinnatifida* could potentially be used as a substrate for the enzymatic synthesis of FO, providing new opportunities for the development of biofunctional materials.

**Figure 2:**
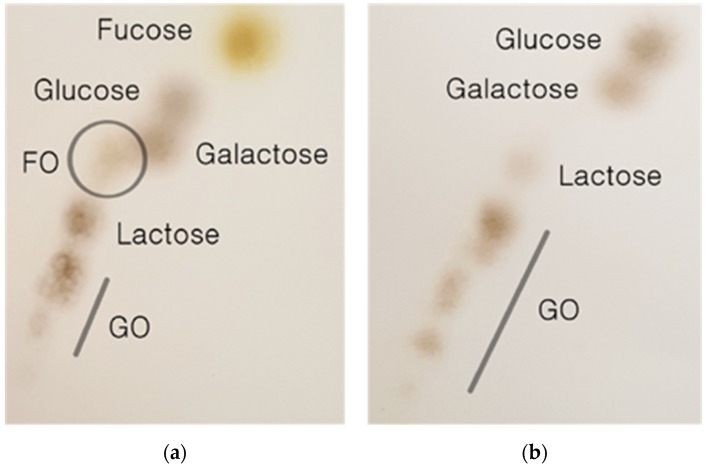
Determination of synthesized oligosaccharides by 2D thin layer chromatography (TLC) using β-galactosidase of *B. longum* RD47. Transglycosylated product was created by combining monosaccharide fraction and lactose as substrate: (**a**) fucosylated oligosaccharide (FO) was newly produced via transglycosylation of fucose in the monosaccharide fraction and lactose; (**b**) as fucose was not present during the β-galactosidase reaction, transglycosylation was not induced and FO production was not observed. GO denotes galactosyl oligosaccharides.

Figure 2 shows that the β-galactosidase of *B. longum* RD47 is able to produce FO from l-fucose monosaccharide fractions and lactose. The TLC profile in Figure 2a shows the result of an enzymatic reaction between the monosaccharide fraction and lactose with β-galactosidase of *B. longum* RD47. In the absence of l-fucose, no FO was produced (Figure 2b).

The newly generated FO and l-fucose contained in the monosaccharide fraction were clearly distinguished from other molecular spots. Figure 2b shows the effect of *B. longum* RD47 β-galactosidase treatment on lactose that did not include the monosaccharide fraction treatment. As l-fucose was not treated, newly generated FO was not observed. Lactose was hydrolyzed into galactose and glucose by β-galactosidase and galactosyl oligosaccharides were formed with various degrees of polymerization, as shown in Figure 2a,b. The Figure 2a TLC profile results are the same as those previously shown, where commercially available l-fucose (American Chemical Society grade, ≥95%) was used as a fucose substrate for the synthesis of FO via *B. longum* RD47 β-galactosidase. The FO spot was previously confirmed to be composed of fucose and galactose by MALDI-TOF and LC-ESI/MS [18]. Taken together, the fucose contained in *U. pinnatifida* sporophyll as a form of fucodian was successfully used for the synthesis of FO by *B. longum* RD47 β-galactosidase.

## 3. Materials and Methods

### 3.1. Extraction of Crude Fucoidan from U. pinnatifida Sporophyll

*U. pinnatifida* sporophyll grown in Wando, Korea was purchased from a local grocery in Pohang, Korea. All reagents were purchased from Sigma-Aldrich (Sigma, St. Louis, MO, USA) unless otherwise noted. The purification of fucoidan from *U. pinnatifida* sporophyll was performed using the Kim et al. method [21], modified as follows: *U. pinnatifida* sporophyll was homogenized with a food grinder and refluxed with a mixture of methanol/chloroform/water 4/2/1 (*v/v/v*) using a rotary evaporator to remove colored matter and phenol compounds prior to extraction [31]. After pretreatment, the mixture was centrifuged at 10,000× *g* for 20 min at 4 °C, after which the supernatant was discarded. The extract was filtered through a cellulose flat sheet filter membrane (Whatman No.1, Whatman, Maidstone, UK). The filtrate was then neutralized with 1 N NaOH, and the solution was precipitated with three volumes of ethanol. After centrifugation at 6,000× *g* for 30 min at 4 °C, the precipitate was dissolved in distilled water. The pH of the suspension was adjusted to 2.0 with 1 N HCl, and CaCl_2_ was added to the final concentration of 2 M. After centrifugation at 6,000× *g* for 30 min at 4 °C, the precipitate was removed. Then, the supernatant was treated with three volumes of ethanol and repeated three times. The final precipitate was dissolved in distilled water and dialyzed through a MWCO 3500 membrane (Spectrum Laboratories of Repligen, Waltham, MA, USA) at 4 °C in distilled water for 48 h and freeze-dried. This product was then designated as crude fucoidan and its yield was calculated as follows:

Yield (%) = [amount of crude fucoidan (g) / amount of *Undaria pinnatifida* sporophyll (g)] × 100.

The extract was further purified by column chromatography. One gram of crude fucoidan was dissolved in 10 ml of distilled water and applied to a DEAE–cellulose column (100 ml) that had been pre-equilibrated with distilled water (pH 7.0 adjusted with 0.1 M NaOH) and eluted with the same buffer containing increasing concentrations of NaCl (0.1, 0.5, 1.0, 1.5, 2.0 M) until no more carbohydrate was detected. Each fraction was assayed for carbohydrates by thin layer chromatography (TLC). The carbohydrate-positive fractions were pooled together and dialyzed for 24 h through an MWCO 3500 membrane in distilled water and freeze-dried. This product was designated purified crude fucoidan.

### 3.2. Preparation of Purified Fucoidan Hydrolysate from Crude Fucoidan

To obtain purified fucoidan hydrolysate, 10 mg of crude fucoidan was dissolved in 1 mL of distilled water, and an equal volume of 0.2 N HCl was added and allowed to stand for 1 h at 120 °C in an autoclave. After this treatment, the mixture was neutralized with 1 M NaOH, filtered through a 0.45 μm syringe filter, and vacuum dried using a Speed-Vacuum (ScanSpeed 40, LaboGene, Lillerød, Denmark). Various ions including sulfate, phosphate, and uronic acid present in the hydrolysate were removed by ion exchange resin on IRA-400 (Chloride Form, Sigma, USA) and DOWEX 50XW4 (Hydrogen form, Sigma, USA) in an open column. One gram of dried hydrolysate was dissolved in 50 mL of distilled water and loaded onto an IRA-400 open column. The column was then eluted by distilled water and fractions were collected and loaded onto a DOWEX 50XW4 open column. The column was eluted by distilled water and fractions were collected and concentrated in a speed vacuum concentrator (ScanSpeed 40, LaboGene, Denmark). Impurities present in hydrolysate were removed by gel permeation chromatography on a PD Miditrap G-10 (5.3 mL, GE Healthcare, Chicago, IL, USA). One milliliter of hydrolysate dissolved in sterilized water was loaded onto a gravity column of PD Miditrap G-10. The column was eluted with 2 mL of sterilized water. Fractions containing monosaccharides were detected by TLC. Finally, fucose-positive fractions were collected and concentrated by a speed vacuum concentrator.

### 3.3. Evaluation of Monosaccharide Composition of Fucoidan Hydrolysate by Bio-LC

Bio-LC was performed to determine the composition of the monosaccharides [32]. Bio-LC analysis was carried out on a Dionex-2500 ion chromatography (Thermo Fisher Scientific, Waltham, MA, USA) instrument equipped with an ED40 Gold electrode, pulsed by an amprometry detector (Thermo Fisher Scientific, Waltham, MA, USA). All samples were microfiltered through a 0.2 μm cutoff PVDF membrane filter, and the sample injection volume was 10 µL. All chromatographic separation procedures were carried out on a CARBOPAC_PA1 column (4 × 250 mm) from Dionex. The 1.0 mL/min flow rate was constant. The mobile phase was 2 mM potassium hydroxide, and the solvent composition was performed as follows: 2 mM (1–35 min); 2–100 mM (35–36 min); 100 mM (36–56 min); 100–2 mM (56–57 min); 2 mM (58–63 min). Standard solutions were prepared in distilled water to calculate the concentration of monosaccharides in the samples.

### 3.4. Synthesis of Fucosylated Oligosaccharide Using B. longum RD47

Bifido LTD (Hongcheon, Korea) generously donated frozen *B. longum* RD47 cell stock. *B. longum* RD47 was activated by two successive precultures in MRS medium (Difco, Detroit, MI, USA) with 0.05% (*w*/*v*) cysteine–HCl at 37 °C for 18 h. The activated *B. longum* RD47 was inoculated in 8 ml MRS containing 0.05% (*w*/*v*) cysteine–HCl and grown at 37 °C for 18 h under anaerobic conditions. The activated microorganisms were centrifuged at 16,000× *g* for 5 min. Cells were harvested by centrifugation and washed twice in 50 mM sodium phosphate buffer (PB, pH 6.6). The supernatant was then discarded. For the preparation of β-galactosidase extracts, washed *B. longum* RD47 cells were resuspended in one volume of PB (pH 6.6) and disrupted with a sonicator (Sonicator 500, Q-Sonica, Newtown, CT, USA) in 1.0 s on/1.0 s off intervals for 5 min. Supernatant was used after centrifugation at 16,000× *g* for 12 min at 4 °C. β-Galactosidase activity was measured by testing the para-nitrophenol (pNP) d-β-galacto-pyranosides as substrate. Enzyme solution (80 μL, 5 μL of crude enzyme extracts in 75 μL of PB) was added to 20 μL of 5 mM pNP-d-galactoside in 50 mM PB (pH 6.6). The mixture was incubated at 37 °C for 10 min and the reaction was stopped by adding 100 μL of 1 M Na_2_CO_3_. Enzyme activity was measured via spectrophotometer in microplates at 405 nm. Specific activity (enzyme activity level relative to cell mass) was determined as units of β-galactosidase activity. One unit, equivalent to the relative enzyme activity, was determined as the amount of product converted by 1 mL of *B. longum* RD 47 crude enzyme over 1 min. To synthesize FO, 1 g of fucoidan hydrolysate and 400 mg of lactose were mixed, 1 mL of which solution in PB (pH 6.6) was prepared for reaction. The enzyme extract (40 μL) was added to a sugar solution and the mixture was incubated at 37 °C. After 24 h incubation, the reaction was terminated by boiling for 10 min. The total activity of β-galactosidase was 5.31 µM pNP (min·mL)^−1^. The types of carbohydrates produced as a result of the enzyme reaction were evaluated by one- or two-dimensional TLC. After enzyme reaction, samples were loaded onto silica gel plate 60 (Merck, Darmstadt, Germany); the mobile phase was composed of 1-propanol, distilled water, and ethyl acetate (7/2/1, *v*/*v*/*v*). The sulfuric acid–ethanol (1/9, *v*/*v*) solution was sprayed and dried and the developing solvent of 2D TLC was composed of ethyl acetate, 1-propanol, DW, and acetic acid (4/2/2/1, *v*/*v*/*v*/*v*). Finally, the silica gel plate was heated at 110 °C for 5 min for visualization.

## 4. Conclusions

In this study, the fucose containing the polysaccharide known as fucoidan was extracted from *U. pinnatifida* sporophyll. The purified fucoidan was hydrolyzed and used to synthesize FOs using a crude enzyme extract from *B. longum* RD47. The synthesis of FO was confirmed by 2D TLC, indicating that fucose from *U. pinnatifida* sporophyll was confirmed as a substitute for expensive commercial l-fucose. For later application, l-fucose from *U. pinnatifida* could be used as a substrate for the enzymatic condensation of FO. This may provide new opportunities for the development of new prebiotics. To the best of our knowledge, this is also the first report to produce prebiotic FO using *Bifidobacterium* spp.
